# Clustered Regularly Interspaced Short Palindromic Repeats-Associated Protein System for Resistance Against Plant Viruses: Applications and Perspectives

**DOI:** 10.3389/fpls.2022.904829

**Published:** 2022-05-26

**Authors:** Fredy D. A. Silva, Elizabeth P. B. Fontes

**Affiliations:** Department of Biochemistry and Molecular Biology/Bioagro, National Institute of Science and Technology in Plant-Pest Interactions, Universidade Federal de Viçosa, Viçosa, Brazil

**Keywords:** CRISPR/Cas, genome editing, resistance to viruses, susceptibility genes, virus-host interactions, plant antiviral immunity

## Abstract

Different genome editing approaches have been used to engineer resistance against plant viruses. The clustered regularly interspaced short palindromic repeats (CRISPR)/CRISPR-associated protein (Cas; CRISPR/Cas) systems to create pinpoint genetic mutations have emerged as a powerful tool for molecular engineering of plant immunity and increasing resistance against plant viruses. This review presents (i) recent advances in engineering resistance against plant viruses by CRISPR/Cas and (ii) an overview of the potential host factors as targets for the CRISPR/Cas system-mediated broad-range resistance and immunity. Applications, challenges, and perspectives in enabling the CRISPR/Cas system for crop protection are also outlined.

## Introduction

The agronomic impact caused by phytopathogens imposes severe yield losses on many important crops worldwide. Agricultural productivity reduction is a recurrent problem due to diseases caused by phytopathogens; viruses are among the principal constraints to crop productivity in a world impacted by accelerated climate change (Savary et al., 2019; Amari et al., 2021). Advances in plant genome editing technology have achieved remarkable breakthroughs in many fields and have been used in plant biotechnology as a tool to improve several traits in an unprecedented way. Part of this progress results from the use of clustered regularly interspaced short palindromic repeats (CRISPR) and CRISPR-associated genes (Cas), CRISPR/Cas system as a tool for genome editing, modulating gene regulation, epigenetic editing, and chromatin engineering (Cong et al., 2013; Doudna and Charpentier, 2014; Adli, 2018; Melo et al., 2021). CRISPR/Cas systems have provided the means to engineer different aspects of the molecular biology’s central dogma (CD) involved in gene regulation, which will undoubtedly accelerate crop improvement (Pramanik et al., 2021a). CRISPR/Cas current applications include gene discovery, introgression, generation of biotic/abiotic stress-resistant crops, plant cell factories, and delayed senescence (Pramanik et al., 2021a). CRISPR/Cas systems have also enhanced the plant immunity and resistance against phytopathogenic viruses by targeting viral genome sequences or host recessive genes in the plant genome (Borrelli et al., 2018; Zahir and Mahfouz, 2021). The first strategy relies on the CRISPR/Cas system harboring sequences that target specific regions of viral genomes. The genome of most plant virus families is composed of RNA; however, some families comprise DNA virus species (Sastry et al., 2019). Based on the genome nature, the plant viruses are classified into 26 families encompassing six different groups: (+) sense ssRNA viruses, (−) sense ssRNA viruses, (+/−) sense ssRNA viruses, dsRNA viruses, (+) sense ssDNA viruses, and (+/−) sense ssDNA viruses. Some economically relevant plant viruses include species from the *Virgaviridae*, *Tospoviridae*, *Geminiviridae*, *Bromoviridae*, and *Potyviridae* families, such as *Tobacco mosaic virus (TMV)*, *Tomato spotted wilt virus (TSWV)*, *Tomato yellow leaf curl virus (TYLCV)*, *Cucumber mosaic virus (CMV)*, *Potato virus Y (PVY)*, *African cassava mosaic virus (ACMV)*, *Plum pox virus (PPV)*, *Brome mosaic virus (BMV)*, and *Bean golden mosaic virus* (BGMV; Rybicki, 2015; Silva et al., 2017). *Geminiviridae* is one large family of plant viruses divided into nine genera bearing agronomic interest because geminivirus species can infect several mono- and dicotyledonous plants, including maize, tomato, potato, cucumber, cassava, pepper, bean, and cotton (Mansoor et al., 2006; Sastry et al., 2019).

Several strategies have been used to control plant viruses. Approaches may be based on traditional techniques, including prophylaxis to prevent virus spread. Other strategies use chemicals to control virus dispersion by natural insect vectors or the removal of infected plants. Additionally, the genomic-assisted selection of resistant cultivars obtained by plant breeding has been used with success (Pérez-de-Castro et al., 2012). More recently, crop transgenic lines expressing small interference RNAs (siRNAs) and RNA interference (RNAi) targeted to viral sequences have been extensively used to obtain resistance (Loriato et al., 2020; Rubio et al., 2020). Successful RNAi-based transgenic plant immunity strategies include the engineered resistance against TYLCV and BGMV (Aragão and Faria, 2009; Leibman et al., 2015). Nevertheless, the emergence of the CRISPR technology with customizable specificities of the RNA-guided nucleases (RGNs), like Cas9, has made targeted genome editing the mainstream method employed by plant virologists to obtain resistance to viruses in several crops (Ali et al., 2015). In addition to simplicity, versatility, and rapid nature, the CRISPR/Cas technology has efficiently modified several viral genomes and endogenous genes in a large variety of crop hosts. CRISPR/Cas-meditated genome interference systems have generated resistance in plants against several viruses, including bean yellow dwarf virus (BeYDV; Baltes et al., 2015), beet severe curly top virus (BSCTV; Ji et al., 2015), tomato yellow leaf curl virus (TYLCV; Tashkandi et al., 2018), African cassava mosaic virus (ACMV; Mehta et al., 2019), cotton leaf curl Multan virus (CLCuMuV; Yin et al., 2019), chili leaf curl virus (ChiLCV; Roy et al., 2019), cauliflower mosaic virus (CaMV; Liu et al., 2018), and cucumber mosaic virus (CMV; Zhang et al., 2018). Although the potential of this strategy is unquestionable, limitations due to off-target editing effects, the rapid evolution of mutants resulting from the mutagenic nature of the CRISPR/Cas system, and the possibility of generating viral escapes in a short time are under constant debate (Mehta et al., 2019). To overcome these issues, new recently discovered CRISPR/Cas systems and multiple gRNAs targeted to different sites have been employed (Shafiq et al., 2021; Zahir and Mahfouz, 2021). In addition, the recessive resistance mediated by potential host susceptibility factors has been considered a promising alternative for applying the CRISPR/Cas system toward broad-range resistance and immunity. This review describes briefly some genome editing tools employed as antiviral strategies and primarily advances in CRISPR/Cas-mediated resistance against plant viruses by targeting viral genomes and/or host susceptibility/recessive resistant genes.

## Molecular Editing Tools to Improve Plant Immunity Against Viruses

Different molecular approaches have been employed to improve plant immunity against viruses. Among those, the nucleases zinc-finger nucleases (ZFNs) and transcription activator-like effector nucleases (TALENs) have taken a prominent place as genome editing techniques (Bibikova et al., 2003; Cheng et al., 2015). ZFNs are fusion proteins of zinc-finger transcriptional activators and *Fok1* endonuclease, whereas, in TALENs, the *Fok1* endonuclease is linked to a bacterial TALE protein. Both designed endonucleases are well-characterized tools for targeting effectors to a specific genome region; they require a specific amino acid sequence (a zinc-finger or TALE motif) that recognizes a DNA sequence of the genome. However, some drawbacks make the use of ZFNs and TALENs limited. Because ZFNs require an amino acid sequence to specify the target site, there is a need for multiple designs to recognize different regions in the genome. Likewise, a single TALE motif recognizes one nucleotide, and hence an array of TALENs is required to associate with longer DNA sequences (Becker and Boch, 2021). Furthermore, in both approaches, the target specificity derives from protein-DNA association; thereby, they can only edit targeted DNA viruses, not being used to edit several plant RNA virus genomes. In addition, replication is partially inhibited for some plant viruses because they can only target a single site (Zahir and Mahfouz, 2021).

**Figure 1:**
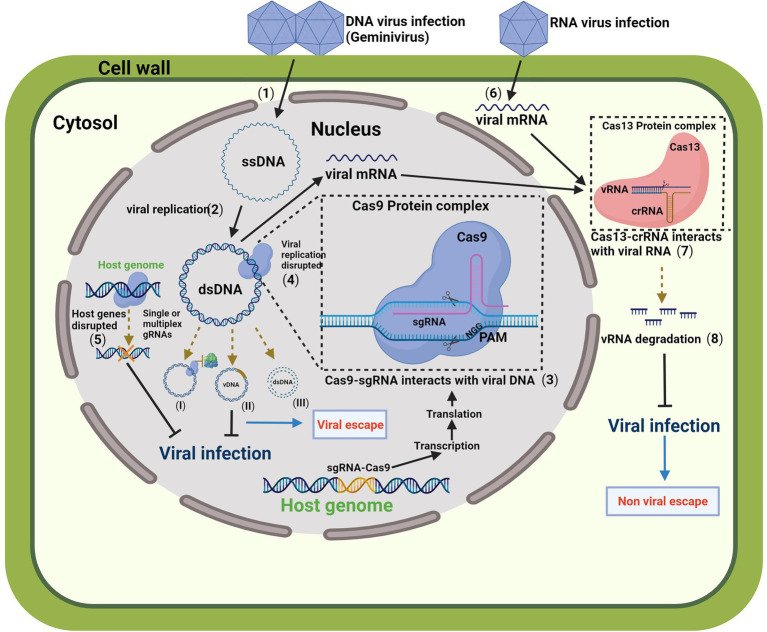
A schematic model for engineering resistance to plant viruses provided by the clustered regularly interspaced short palindromic repeats (CRISPR)/CRISPR-associated protein (CRISPR/Cas) systems. Plant genome transformed with CRISPR/Cas9 system expresses a functional Cas9 protein complex Cas9/gRNA. After Geminivirus infection (1), the viral single-stranded DNA (ssDNA) is delivered into the cytoplasm and translocated to the nucleus. The host nuclear machinery assists the complementary strand synthesis resulting in the viral replication to double-stranded DNA (dsDNA) (2), producing multiple viral copies. The Cas9 protein complex Cas9/gRNA binds to the viral genome (3), which is assisted by a short sequence of 20 nucleotides that directs the Cas9 endonuclease to a specific region of the genomic DNA where it acts as a molecular scissor. A protospacer adjacent motif (PAM) is required. The action of Cas9 results in a double-strand break, and virus replication is disrupted by preventing access to replication proteins (4; I), introducing pinpoint mutations in the viral genome (II), or disrupting the genome by cleavage of dsDNA (III). The CRISPR/Cas system mutagenic property may generate some viral variants. Alternatively, the Cas9 protein complex with multiple gRNAs can target plant host factors to disrupt genes important for viral replication or movement (5). Combining multiplex CRISPR/Cas systems, such as Cas13 and Cas9, is a possible alternative to avoid viral escapes and targeting RNA viruses. After RNA virus infection (6), the viral mRNA interacts with CRISPR/Cas13 system through a short CRISPR RNA (crRNA; 7). The Cas13-crRNA complex is RNA-guided RNA-targeted, and the cleavage of the vRNA induces vRNA degradation (8) and disrupts viral infection. The figure was created with BioRender.com. Cas, CRISPR-associated; CRISPR, clustered regulatory interspaced short palindromic repeats; dsDNA, double-stranded DNA; ssDNA, single-stranded DNA; sgRNA, single guide RNA; vDNA, viral DNA; CRNA, CRISPR RNA; ssRNA, single-stranded RNA; tracrRNA, trans-activating crRNA; and PAM, protospacer adjacent motif.

The CRISPR/Cas system is not limited as ZFNs and TALENs. Due to the feasibility of its mechanism, CRISPR/Cas has become an alternative tool for controlling viral infections by directly editing viral genomes or host factors. CRISPR/Cas was first described as an immune system of archaea and bacteria for defense against viruses by specific interaction of short-viral sequences based on complementarity (Labrie et al., 2010). The system consists of an RNA sequence complementary to the target sequence known as the spacer or CRISPR RNA (crRNA), and a scaffold sequence followed by the CAS protein known as the trans-activating crRNA (tracrRNA; Deltcheva et al., 2011; Gardiner et al., 2022). The most used system is the CRISPR/Cas9 (Figure 1). The mechanism requires a single guide (sg)RNA containing a fusion of 20 nucleotide spacer and scaffold sequence that directs the Cas9 endonuclease to a specific region of the genomic DNA. Additionally, a short NGG sequence and a protospacer adjacent motif (PAM) are required. Cas9 promotes a double-strand break that will be repaired by the host cell resulting in an insertion or deletion that can potentially disrupt the open reading frame of the targeted gene (Doudna and Charpentier, 2014; Jiang and Doudna, 2017).

A recently developed system, the PAMless SpCas9 variant with relaxed nucleotide preference, has overcome these sequence limitations by increasing the number of possible CRISPR/Cas targets (Walton et al., 2020; Ren et al., 2021). Advantages and applications of the CRISPR/Cas systems include (i) improvement of plant immunity by targeting RNA or DNA viral genome with single or multiplex targets (Hussain et al., 2018), (ii) engineering recessive resistance by editing CRISPR/Cas-targeted host-factors required for viral replication or movement (Cao et al., 2020), (iii) recovery of plants with viral symptoms after infection, as a diagnostic system in plants, and (iv) also the generation of edited non-transgenic crops (Aman et al., 2020).

## CRISPR/Cas-Mediated Virus Genome Editing to Control Infection in Plants

Plant viruses are the most diverse phytopathogens globally, impacting cultivated crops. Due to the simplicity of their genome, composed mainly of RNA, plant viruses evolve rapidly. The CRISPR/Cas system can mediate genome interference in DNA or RNA genomes, providing an efficient strategy to control plants viruses (Aman et al., 2020). Accordingly, the CRISPR/Cas9 system has generated plant immunity against viruses in several crops, including beans, tomato, cassava, cotton, chili, wheat, cucumber, and soybean. *Nicotiana benthamiana* plants expressing Cas9-sgRNA to the targeted bean yellow dwarf virus (BeYDV) genome displayed reduced virus load and symptoms (Baltes et al., 2015). Likewise, tomato plants expressing Cas9-sgRNA targeting TYLCV coat protein (CP) or replicase (Rep) sequences were resistant to TYLCV (Tashkandi et al., 2018). Recently, the use of the CRISPR/Cas9 system with multiple sgRNAs, which target essential conserved regions for replication of viral genomes, has improved the resistance of plants against several viruses, including cotton leaf curl Multan virus (CLCuMuV; Yin et al., 2019), wheat dwarf virus (WDV; Kis et al., 2019), and soybean mosaic virus (SMV; Zhang et al., 2020). Likewise, the expression of Cas9-sgRNA targeting ACMV transcription activator (AC2) and replication enhancer (AC3) sequences generated moderate resistance to the begomovirus in cassava (Mehta et al., 2019). Limitations of the CRISPR/Cas9 systems targeting viral genomes include the possibility of generating viral escapes and variants capable of replication (Mehta et al., 2019; Aman et al., 2020). In addition to CRISPR/Cas9, other CRISPR/Cas variants, including Cas3, Cas9, Cas12, Cas13, and Cas14, have potentially been deployed to improve plant immunity (Figure 1). For example, CRISPR/Cas3 system can be used as multiplex targets for double-stranded DNA viruses (dsDNA) or RNA viruses using multiplex sites (Aman et al., 2020). In another study, plants have been engineered using the CRISPR/Cas13 system that targeted TMV and turnip mosaic virus (TuMV) genomes, enhancing plant immunity against these plant RNA viruses (Aman et al., 2018). Furthermore, CRISPR/Cas13 was able to protect potato plants from potato virus Y (PVY; Zhan et al., 2019).

The CRISPR/Cas12 is another system used for resistance to viruses. CRISPR/Cas12 can target both dsDNA and ssDNA viruses (Aman et al., 2020). Applications of CRISPR/Cas12a system include the detection of plant viruses in different crops such as apples and tomato (Cho et al., 2016; Aman et al., 2020; Mahas et al., 2021). The technology has advanced with engineered Cas nucleases to improve their efficiency and precision for the next generation CRISPR editing technologies. The engineered nuclease CRISPR-MAD7 system, a Class 2 type V-A CRISPR-Cas (Cas12a/Cpf1) with low homology to canonical Cas12a nucleases, is a typical example of these new nucleases (Liu et al., 2020). CRISPR/Cas14a is a compact nuclease isolated from archaea, which can be targeted to a single-stranded DNA (ssDNA) genome. The sequence-independence and unrestricted cleavage mechanism make CRISPR/Cas14a a potential tool for engineering resistance against plant ssDNA viruses (Khan et al., 2019). Finally, an alternative strategy employs a combination of CRISPR/Cas systems (Cas3, Cas9, Cas12, Cas13, and Cas14) as a multiplex to enhance plant immunity. These advances in molecular technologies make CRISPR/Cas a powerful tool for improving plant immunity against viruses.

## CRISPR/Cas Systems-Mediated Host Genome Editing to Improve Plant Immunity Against Plant Viruses

Plant viruses are obligate intracellular parasites that require the host cellular machinery to translate their viral genome, replicate, and spread to neighbor cells (Kumar, 2019). Many plant host factors are crucial for viral infections and have been extensively studied as potential targets for controlling plant diseases. Indeed, recessive resistance can be achieved either by silencing a negative regulator of plant defense or a host gene essential for infection. For resistance to viruses, the latter has predominated and been identified as loss-of-susceptibility mutants. The first identified natural recessive resistant genes against RNA viruses mapped to mutations in eukaryotic translation initiation factors *eIF4E* and *eIF4G* genes (Calil and Fontes, 2017). Due to its simplicity and accuracy, the CRISPR/Cas systems have been used as a powerful tool to mediate host genome editing and improve plant immunity against plant viruses in several crops (Table 1). CRISPR/Cas9 sgRNA targeting N′ and C′ termini of eukaryotic translation initiation factor *eIF4E* gene has induced broad-spectrum resistance against the potyviruses zucchini yellow mosaic virus (ZYMV) in cucumber, papaya ringspot mosaic virus-W (PRSV-W) in papaya, and immunity to ipomovirus cucumber vein yellowing virus (CVYV; Chandrasekaran et al., 2016). Due to the physiological importance of translation, the induction of specific pinpoint mutations using CRISPR/Cas is a strategy to avoid deleterious effects by mutating translation initiation genes. Sequence-specific mutations of *eIF(iso)4E* from *Arabidopsis thaliana* by CRISPR/Cas9 provided resistance to TuMV (Pyott et al., 2016). CRISPR/Cas9 editing *eIF4G* in rice has induced resistance to rice tungro spherical virus (RTSV; Macovei et al., 2018). The CRISPR/nCas9 cytidine deaminase system introduced a single mutation in the *eIF4E1* generating resistant plants to clover yellow vein virus (ClYVV; Bastet et al., 2019). Simultaneous CRISPR/Cas9-mediated editions of eIF4E isoforms nCBP-1 and nCBP-2 reduced cassava brown streak virus (CBSV) symptoms and severity (Gomez et al., 2019). In addition to translation initiation factors as targets for CRISPR/Cas-mediated resistance to RNA viruses, the nuclear protein coilin, and flavanone-3-hydroxylase (*F3H*)/flavone synthase II (*FNSII*) genes have also been used as targets for resistance to RNA virus of the *Potyviridae* family. Editing coilin by the CRISPR/Cas9 system increased the resistance of edited potato lines to PVY (Makhotenko et al., 2019). Also, the CRISPR/Cas9 mediated multiplex gene-editing technology has been employed to target flavone-3-hydroxylases [*Glycine max* (Gm)*F3H1* and *GmF3H2*] and flavone synthase II (*GmFNSII-1*) genes as a metabolic engineering approach that resulted in increased isoflavone content and enhanced resistance of edited soybean plants to soybean mosaic virus (SMV; Zhang et al., 2020).

**Table 1:** Summary of CRISPR/Cas system mediating resistance to plant virus by targeting host factors.

CRISPR/Cas system	Plant species	Target host factor	Genus/Plant virus	References
CRISPR/Cas9	Cucumber (*Cucumis sativus* L.)	Host factor eukaryotic translation initiation factor 4E (eIF4E)	*Potyvirus*/*Cucumber vein yellow virus* (CuVYV)	Chandrasekaran et al., 2016
CRISPR/Cas9	Cucumber (*Cucumis sativus* L.)	Host factor eukaryotic translation initiation factor 4E (eIF4E)	*Potyvirus*/*Zucchini yellow mosaic virus* (ZYMV)	Chandrasekaran et al., 2016
CRISPR/Cas9	Cucumber (*Cucumis sativus* L.)	Host factor eukaryotic translation initiation factor 4E (eIF4E)	*Potyvirus*/*Papaya ring spot virus-W* (PRSV-W)	Chandrasekaran et al., 2016
CRISPR/Cas9	*Arabidopsis thalaiana*	Host factor eukaryotic translation initiation factor eIF(iso)4E	*Potyvirus*/*Turnip mosaic virus* (TuMV)	Pyott et al., 2016
CRISPR/Cas9	Rice (*Oriza sativa*)	Host factor eukaryotic translation initiation factor eIF4G	*Tungrovirus*/*Rice tungo spherical virus* (RTSV)	Macovei et al., 2018
CRISPR/nCas9 cytidine deaminase	*Arabidopsis thaliana*	Substitutions encoded by a *Pisum sativum* eIF4E virus-resistance allele into the *Arabidopsis thaliana* eIF4E1	*Potyvirus*/*Clover yellow vein virus* (RTSV)	Bastet et al., 2019
CRISPR/Cas9	Cassava (*Manihot esculenta* Crantz)	Simultaneous editions of IF4E isoforms nCBP-1 and nCBP-2	*Ipomovirus*/*Cassava brown streak virus* (CBSV)	Gomez et al., 2019
CRISPR/Cas9	Potato (*Solanum tuberosum*)	Nuclear Coilin	*Potyvirus*/*Potato virus Y* (PVY)	Makhotenko et al., 2019
CRISPR/Cas9	Soya bean [*Glycine max* (L.) Merr.]	Multiple targets of isoflavanoids pathway flavone-3-hydrolases (*GmF3H1*, *GmF3H2*) flavone synthase II (*GmFNSII-1*)	*Potyvirus*/*Soya bean mosaic virus* (SMV)	Zhang et al., 2020
CRISPR/Cas9	Tomato (*Solanum lycopersum*)	Susceptibility (*S*-gene) factor	*Begomovirus*/*Tomato yellow leaf curl virus* (TYLCV)	Pramanik et al., 2021b

Despite these reports, the application of CRISPR/Cas for host genome editing in plant immunity has been limited because of the restricted repertoire of characterized naturally loss-of-susceptibility mutants or recessive-resistant genes. In the lack of known recessive resistant genes, a loss-of-function mutation in susceptibility genes, which will not cause deleterious effects on plant growth and productivity, can be an alternative target for CRISPR/Cas-mediated host immunity. In fact, the inactivation of a necessary host factor for infection is supposed to account for recessively inherited disease resistance to plant viruses. For the ssDNA bipartite begomoviruses, two susceptibility genes, the endosomal NSP-interacting syntaxin-6 domain-containing protein (NISP), and NSP-interacting GTPase (NIG), which are involved in the intracellular traffic of viral DNA, may be targets for enhancing resistance in crops (Carvalho et al., 2008; Gouveia-Mageste et al., 2021). Silencing of *NISP* enhanced resistance to cabbage leaf curl virus (CabLCV) in Arabidopsis without yield penalty, an essential property for considering susceptibility genes as targets for engineering recessive resistance. Accordingly, the silenced lines display lower DNA viral load and attenuated symptoms and are phenotypically indistinguishable from the control lines under normal conditions (Gouveia-Mageste et al., 2021). Likewise, CRISPR/Cas9 sgRNA has been employed to target the susceptibility gene (S-gene) *SIPelo* for the monopartite begomovirus TYLCV inducing resistance in edited tomato plants against the virus (Pramanik et al., 2021b). Silencing the S-gene suppressed viral DNA accumulation and restricted the systemic spread of TYLCV to non-inoculated leaves (Pramanik et al., 2021b). Collectively, these results demonstrate the potential of CRISPR/Cas systems to generate host-mediated immunity to DNA and RNA viruses by targeting susceptibility genes or resistant recessive genes. The efficiency of the CRISPR/Cas systems in introducing mutagenesis in multiple target sites offers a precise genome editing technology for engineering a variety of transgene-free resistant crops.

## Conclusion and Future Perspectives

Clustered regularly interspaced short palindromic repeats/Cas systems have a central role in plant biotechnology as an accurate molecular tool for editing genomes, rapidly improving desired traits, creating new plant varieties, and enhancing plant immunity against phytopathogens. The use of CRISPR/Cas systems is suitable for mediating viral genome editing while maintaining the biological functions of cells. Additionally, CRISPR/Cas systems have the potential to edit host factors, improving plant immunity against plant viruses. Nevertheless, a drawback in CRISPR/Cas-mediated host genome editing to enhance plant immunity is the limited repertoire of well-characterized recessive resistant genes or host susceptibility genes in which mutations are not likely to cause host growth defects. A better understanding of the host-virus interactome will expand the use of CRISPR/Cas for editing host susceptibility genes, which may be more efficient targets for durable resistance against viruses.

Meanwhile, advances in the next generation CRISPR editing technology variants, such as the CRISPR-MAD7 system and engineered nucleases Cas12a, increase the accuracy, range of possibilities, and applications. Engineered Cas MAD7-RR, MAD7-RVR, and M-AFID (MAD7-APOBEC fusion-induced deletion) increase the targeting range of MAD7 by creating predictable deletions from 5′-deaminated Cs to the MAD7-cleavage site. This new CRISPR-MAD7 system has an efficiency of up to 65.6%, as demonstrated in mutant rice and wheat plants (Lin et al., 2021). MAD7 can expand the CRISPR toolbox for genome engineering due to its highly efficient target to gene disruption and insertions, different protospacer adjacent motifs, and small-guide RNA requirements (Liu et al., 2020). Other advances in CRISPR/Cas systems have improved precision and provided multiple edited sites in viral genomes toward reaching a lower risk of generating viral escapes or new variants. Using new CRISPR/Cas systems (Cas3, Cas12, Cas13, and Cas14) as multiplex sgRNAs targeting different sites is a new and more efficient strategy to improve broad-spectrum resistance, prevent viral infections, and control disease in the field.

## Author Contributions

FDAS and EPBF wrote the drafts with input from both authors. All authors contributed to the article and approved the submitted version.

## Funding

This work was partially supported by the Council for Advanced Professional Training (CAPES)—88887.511855/2020-00, National Council for Scientific and Technological Development (CNPq)—441955/2019-3, and Fapemig.

## Conflict of Interest

The authors declare that the research was conducted in the absence of any commercial or financial relationships that could be construed as a potential conflict of interest.

## Publisher’s Note

All claims expressed in this article are solely those of the authors and do not necessarily represent those of their affiliated organizations, or those of the publisher, the editors and the reviewers. Any product that may be evaluated in this article, or claim that may be made by its manufacturer, is not guaranteed or endorsed by the publisher.
